# Audit and feedback to reduce unwarranted clinical variation at scale: a realist study of implementation strategy mechanisms

**DOI:** 10.1186/s13012-023-01324-w

**Published:** 2023-12-11

**Authors:** Mitchell Sarkies, Emilie Francis-Auton, Janet Long, Natalie Roberts, Johanna Westbrook, Jean-Frederic Levesque, Diane E. Watson, Rebecca Hardwick, Kim Sutherland, Gary Disher, Peter Hibbert, Jeffrey Braithwaite

**Affiliations:** 1 Australian Institute of Health Innovation, Macquarie University, Sydney, Australia; 2 School of Health Sciences, University of Sydney, Sydney, Australia; 3 Centre for Primary Health Care and Equity, University of New South Wales, Kensington, NSW Australia; 4 NSW Agency for Clinical Innovation, Sydney, Australia; 5 Bureau of Health Information, St Leonards, NSW Australia; 6 Peninsula Medical School, Faculty of Health, University of Plymouth, Plymouth, UK; 7 NSW Ministry of Health, Sydney, Australia; 8 Allied Health and Human Performance, IIMPACT in Health, University of South Australia, Adelaide, SA Australia

**Keywords:** Realist evaluation, Implementation science, Low-value care, Quality of health care, Clinical audit, Nursing audit, Medical audit, Clinical competence, Practice guideline, Evidence-based practice

## Abstract

**Background:**

Unwarranted clinical variation in hospital care includes the underuse, overuse, or misuse of services. Audit and feedback is a common strategy to reduce unwarranted variation, but its effectiveness varies widely across contexts. We aimed to identify implementation strategies, mechanisms, and contextual circumstances contributing to the impact of audit and feedback on unwarranted clinical variation.

**Methods:**

Realist study examining a state-wide value-based healthcare program implemented between 2017 and 2021 in New South Wales, Australia. Three initiatives within the program included audit and feedback to reduce unwarranted variation in inpatient care for different conditions. Multiple data sources were used to formulate the initial audit and feedback program theory: a systematic review, realist review, program document review, and informal discussions with key program stakeholders. Semi-structured interviews were then conducted with 56 participants to refute, refine, or confirm the initial program theories. Data were analysed retroductively using a context-mechanism-outcome framework for 11 transcripts which were coded into the audit and feedback program theory. The program theory was validated with three expert panels: senior health leaders (*n* = 19), Agency for Clinical Innovation (*n* = 11), and Ministry of Health (*n* = 21) staff.

**Results:**

The program’s audit and feedback implementation strategy operated through eight mechanistic processes. The strategy worked well when clinicians (1) felt ownership and buy-in, (2) could make sense of the information provided, (3) were motivated by social influence, and (4) accepted responsibility and accountability for proposed changes. The success of the strategy was constrained when the audit process led to (5) rationalising current practice instead of creating a learning opportunity, (6) perceptions of unfairness and concerns about data integrity, 7) development of improvement plans that were not followed, and (8) perceived intrusions on professional autonomy.

**Conclusions:**

Audit and feedback strategies may help reduce unwarranted clinical variation in care where there is engagement between auditors and local clinicians, meaningful audit indicators, clear improvement plans, and respect for clinical expertise. We contribute theoretical development for audit and feedback by proposing a Model for Audit and Feedback Implementation at Scale. Recommendations include limiting the number of audit indicators, involving clinical staff and local leaders in feedback, and providing opportunities for reflection.

**Supplementary Information:**

The online version contains supplementary material available at 10.1186/s13012-023-01324-w.

Contributions to the literature

- The effectiveness of audit and feedback on unwarranted clinical variation is influenced by the individual strategy components used and how they interact with local contextual circumstances.
- This uncertainty around what works, for whom, and why, limits the ability to design audit and feedback strategies that lead to the greatest impact when implemented at scale.
- We demonstrated that audit and feedback may impact unwarranted clinical variation through eight potential mechanisms, which are only triggered under certain contextual circumstances.
- We developed a Model for Audit and Feedback Implementation at Scale, which advances our understanding of how audit and feedback can be implemented across entire health systems at scale.

## Background

Addressing unwarranted clinical variation in hospital care remains a key challenge to health system improvement. Despite all the improvements to health outcomes from modern medicine, care is not always provided in line with clinical practice guidelines [1, 2]. The underuse of effective services withholds potential beneficial outcomes from patients [3, 4]. Overuse or relying on insufficient and outdated evidence can waste valuable resources [5–8]. Misuse still befalls some patients who experience iatrogenic harm or adverse events [9]. These variations in care are considered unwarranted when they differ disproportionately from available evidence or the informed choices of patients [10, 11].

Clinical variation may occur due to a multitude of complex and interacting reasons. Warranted variation refers to situations where multiple effective options exist, and choice depends on patient preference. Sometimes there is genuine uncertainty among clinicians [12–16] in different contextual circumstances [17, 18] due to the unavailability of objective criteria to define appropriate care [19, 20]. However, when these situations arise in practice, medical opinion can tend to influence treatment choice [21–23]. Unwarranted variation is a value judgement about whether this variation is appropriate. Decisions to deviate from guideline recommendations can be influenced by clinician beliefs [24], preferences, or financial incentives [25, 26]. The evidence is well established that healthcare overuse and underuse may compromise patient care, increase inefficiency, and contribute to healthcare inequality [27]. Therefore, opportunities to change practice in a way that meets patients’ needs, according to clinical practice guidelines, are garnering the attention of health system decision-makers.

Audit and feedback is a common strategy used to reduce unwarranted clinical variation [28]. Providing healthcare professionals with performance feedback relative to specific target indicators may prompt modifications to their practice. Small improvements in provider compliance from audit and feedback have been demonstrated (median 4.3%); however, the range of effects between studies is substantial (− 9% to 70%) [29]. This wide variability indicates that not all audit and feedback strategies are optimally designed, operationalised, and components adequately specified. Many have not considered the previously hypothesised mechanisms for how audit and feedback works or can be bundled with other co-interventions [30, 31]. Furthermore, the success of audit and feedback strategies can be influenced by local contextual circumstances, in addition to the individual strategy components themselves [32]. Brown et al., developed the Clinical Performance Feedback Intervention Theory, which suggests that effective feedback is cyclical and sequential; becoming less effective if any one process within the cycle fails [32]. According to this theory and supporting empirical evidence, feedback interventions are more effective when they provide training and support regarding feedback [33], where there are health professionals with skills and experience in quality improvement, and the clinical topic under focus is targeted [33, 34]. Greater understanding of how and why audit and feedback work (or does not work) in different circumstances, is required to design better strategies.

### Implementation context

In Australia, the New South Wales (NSW) Health system has invested in several programs to operationalise the concept of “value-based healthcare”. Value in health is defined as the net improvement of welfare across individuals within a society [35]. A value-based healthcare system is thus characterised by the optimisation of the welfare derived from health services including common or shared goals for equity and fairness, considering that resources used to achieve benefits in one manner are the same resources that can no longer be used for other purposes. Value-based healthcare is operationalised in NSW Health through several state-wide programs [36]. Leading Better Value Care (LBVC) is one flagship program administered by the NSW Ministry of Health, in partnership with the Agency for Clinical Innovation, Clinical Excellence Commission, and Cancer Institute NSW, to implement models of care for specific chronic conditions state-wide at scale. Three of the first eight LBVC initiatives focussed on reducing unwarranted variation in inpatient hospital care for patients with chronic obstructive pulmonary disease (COPD), chronic heart failure (CHF), and diabetes mellitus. These patient cohorts were selected due to high and persisting admission, readmission and complication rates, including small area and facility-level variation [37, 38]. Solutions included new or improved models of care, clinical audits, improved documentation, and patient coding processes [36]. A state-wide audit and feedback strategy was undertaken by the Agency for Clinical Innovation, delivered at the level of each hospital using medical record review and group feedback sessions to identify areas of practice to target with a quality improvement plan. The program was implemented across more than 100 facilities in the 2017–2018 financial year and presents a unique natural experiment to examine how and why the audit and feedback implementation strategy impacted unwarranted variation in care (Table 1). The initial COPD, CHF, and diabetes mellitus program logic are provided in Additional files 1 and 2.

**Table 1:** Description of the three LBVC initiatives and audit and feedback implementation strategy targeting inpatient variation in care

Initiative	Clinical priorities	Objectives	Implementation strategy
Chronic heart failure and chronic obstructive pulmonary disease	• Timely cardiology review and access to investigations • Evidence-based pharmacological treatment, fluid management, and oxygen therapy • Spirometry to confirm and assess severity of COPD exacerbation • Delivery of oxygen and non-invasive ventilation • Timely referral to a multidisciplinary heart failure management program or pulmonary rehabilitation; standardised communication to support transfer to the community; identification of advanced heart failure and COPD for palliative care	• Improve health outcomes and efficient service delivery • Reduce unwarranted clinical variation • Optimise patient and carer experience • Increase the education, resources and support provided to people	• Local clinicians and managers review practice and implement strategies to align routine care with best practice • A range of responses is expected and encouraged. Localised improvement plans monitored • Systematisation of local processes to detect and address unwarranted clinical variation
Inpatient management of diabetes mellitus	• BGL^a^ test taken in the emergency department and a current HbA1c recorded early in the medical file • A minimum of four BGL checks in the first 24 h of admission, and regular BGL monitoring for patients requiring insulin. A basal-bolus-supplemental insulin regimen is considered for all patients requiring subcutaneous insulin • Timely and appropriate access to specialist care if required • A diabetes management plan with standardised communication to support transfer for ongoing management	• Provide support for audit and feedback, continuous improvement, and benchmarking • Increase identification of people with diabetes in hospitals who require insulin • Increase clinical staff knowledge and skills to provide best-practice care • Facilitate access to specialised diabetes care • Reduce insulin prescribing errors • Reduce hyper- and hypo-glycaemic episodes and other insulin-related adverse events • Reduce complication rates for people with diabetes requiring insulin • Reduce hospital length of stay for people with diabetes who require insulin • Improve the patient and carer experience	• A capability-building strategy to support best practice management of people with diabetes who require insulin including implementation of a subcutaneous insulin chart • Define best practice management of people in hospitals with diabetes who require insulin • Advice and support for local audits to support feedback, continuous improvement, and benchmarking

Sourced from the NSW Agency for Clinical Innovation’s Monitoring and Evaluation Plans for the LBVC initiatives [37, 38]

^a^*BGL* blood glucose level

*COPD* chronic obstructive pulmonary disease

### Aim

The aim of this study was to identify how, why, and in what contexts the audit and feedback strategy contributed to the implementation of a state-wide value-based healthcare program to reduce unwarranted variation in care at scale. The main objectives were to:

1. Identify and articulate the audit and feedback implementation strategies used to reduce unwarranted variation in care for patients with CHF, COPD, and diabetes.
2. Determine the mechanisms (underlying social, cultural, structural, individual, and relational processes or events [39, 40]) by which the strategies operated to produce desired or undesired effects.
3. Investigate the impact of different contextual circumstances on the relationship between these implementation mechanisms and outcomes of interest.

## Methods

### Study design and rationale

A realist study was conducted to investigate how the LBVC audit and feedback strategy contributed to the implementation of a state-wide value-based healthcare program to reduce unwarranted variation in care. Realist study designs are theory-driven evaluations, based on a realist philosophy of science [41, 42]. They focus on generating plausible explanations for how and why programs work under different circumstances, referred to as “program theories” [43–45]. Therefore, the social responses to programs are considered the primary mechanisms of change and focus of inquiry, rather than the programs themselves [39, 46, 47]. This approach is well suited to address our objective to determine the mechanisms by which audit and feedback operate to produce desired or undesired effects. A greater understanding of these explanations may enable the replication of success, and avoid unintended outcomes when implementing healthcare improvement programs at scale [48–50].

**Fig. 1:**
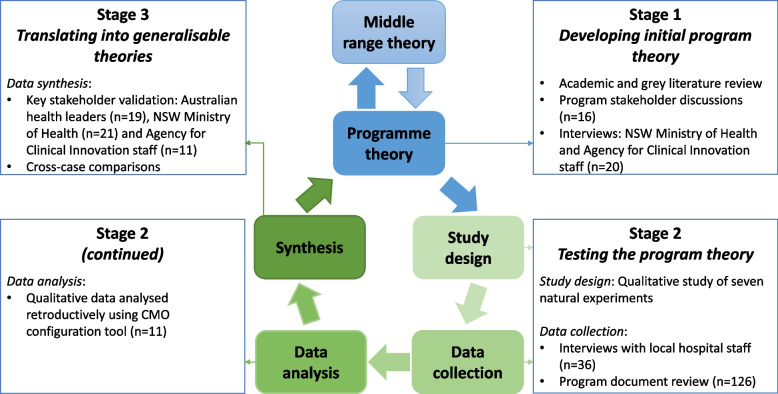
Realist study stages. CMO = context–mechanism–outcome.  Adapted from Pawson et al. [42] and Sarkies et al. [51, 54]

The study was conducted and reported according to our published protocol [51] and the RAMESES II (Realist And Meta-narrative Evidence Syntheses: Evolving Standards) reporting standards for realist studies [52] (Additional file 3). Ethical approval was provided by Macquarie University (Project ID 23816) and Hunter New England Human Research Ethics Committees (Project ID 2020/ETH02186). Three stages of research took place to posit, test, and refine program theories for the audit and feedback implementation strategy to reduce unwarranted variation in care (Fig. 1) [53]. In stage 1, initial program theories were identified for how the audit and feedback strategy was implemented to reduce unwarranted variation in care; in stage 2, these hypothesised program theories were then tested and refined; and in stage 3, the program theories were translated into generalisable implementation models. Multiple data sources were used across different time points to ensure the information best placed to inform the study was captured. Study protocol adaptations are described in Additional file 4.

### Setting

This realist study took place in NSW, Australia, examining the implementation of the LBVC program at scale between 2017 and 2021. In Australia, state and territory governments are responsible for healthcare planning and delivery by public hospitals. NSW Health provides universal access to health care services for Australia’s most populous state (~ 8.15 million people in 2022), which is operated by more than 130,000 staff spread across 234 public hospitals and multi-purpose facilities, over a geographical area of more than 800,000 km^2^ [36]. In relation to clinical care and quality improvement during the COVID-19 pandemic, throughout 2020 to 2021 NSW experienced among the lowest rates of COVID-19 internationally. In 2020, there were 4,782 confirmed cases of COVID-19 in NSW. In each month during the first half of 2021, there were few to no community-acquired cases. After the emergence of the Delta variant in June and Omicron in November 2021, there were a total of 280,601 confirmed cases in the second half of 2021 [55].

### Participant recruitment and sampling

A maximum diversity, purposive sampling approach was taken to obtain a variety of organisational and individual perspectives considered best placed to provide information on the implementation of the initiatives. NSW Ministry of Health, Agency for Clinical Innovation, and local hospital staff eligible for participation were identified initially by the project partners and invited by the partners via email. Further respondents well placed to discuss the proposed program theories were identified through snowballing and were approached by the investigators directly via email. Informed consent was recorded verbally at the commencement of each interview.

### Data collection and analysis

#### Stage 1: identifying the initial program theory

We developed and undertook a Realist Dialogic Approach to identify the initial program theory for the audit and feedback strategy to reduce unwarranted variation in care [53]. A program theory explains how and why a program is expected to work and makes an explicit connection between the activities undertaken in the program and the expected outcomes from those activities. The Realist Dialogic Approach process followed four phases to (1) understand relevant theories, (2) review academic and grey literature, (3) conduct informal discussions with key stakeholders, and (4) undertake research-group conversations. First, several sources of academic and grey literature were reviewed to identify hypotheses that could inform the development of an initial program theory for how and why the program was expected to work. This included a systematic review of studies examining implementation determinants for chronic condition hospital avoidance programs (13 articles) [45] and realist review conceptualising contexts, mechanisms, and outcomes for implementing large-scale, multisite hospital improvement initiatives (*n* = 51 articles) [56] to identify initial program theory propositions. The systematic review and realist review formed an initial source of data to identify theory propositions that could then be situated within the LBVC program using other sources of data. Second, public documents pertaining to the LBVC program were reviewed to modify and situate these propositions within the LBVC program of interest. Once these theory propositions were made specific to the LBVC program, informal discussions with key program stakeholders (~ 16 stakeholders) were also used to map any differences between how the implementation was planned and how it was operationalised in practice. The initial program theory, in the form of context-mechanism-outcome configurations and interview question guides, is provided in Additional file 5.

#### Stage 2: testing the hypothesised program theory

Internal documents pertaining to the LBVC program (126 documents) were reviewed to further situate the initial program theories within the program of interest. Semi-structured realist interviews [57, 58] to refute, refine, or confirm the initial program theories were then conducted with 56 participants via videoconference or telephone at the participants’ preferred time and location. The interviews were 30–60 min in duration, audio recorded and transcribed verbatim. They were conducted by two experienced researchers (MNS, EF-A) using an interview guide that was pilot-tested prior to data collection. The interviews sought to build a nuanced understanding of how the audit and feedback strategy was used to implement the program. We invited the participants to clarify or modify our hypothesised descriptions of the program implementation from their experience [59]. Data sources are summarised in Table 2.

**Table 2:** Summary of data sources

Data source	Number of sources
Systematic review, *n* articles	13
Realist review, *n* articles	51
Program stakeholder informal discussions, *n* stakeholders	16
Program documentation review, n documents	126
Interviews, *n* participants:	
NSW Ministry of Health and Agency for Clinical Innovation	20
Local hospital	36

Retroductive analysis was undertaken by five investigators (MNS, EF-A, CP, NR, JCL) concurrently with data collection using NVivo 20. This analysis used both inductive and deductive logic to identify causal mechanisms behind patterns of change. Investigators cycled between inductive and deductive logic across the program documents and interview transcripts, incorporating their own insights [60]. Transcripts were read in full and coded line-by-line according to a context-mechanism-outcome configuration framework [61–63]. Data were coded when links between the (1) contextual circumstances required to trigger change, (2) mechanisms generating change processes, and (3) outcomes produced when mechanisms are triggered in certain contextual circumstances for audit and feedback were identified [42, 64]. The quotes were categorised as either supporting, refuting, or refining the context-mechanism-outcome configuration. Memos were used to record the decision-making process according to processes outlined by Gilmore et al. [60]. Approximately 20% of the coded transcripts were checked by a second investigator and any disagreements between the investigators were resolved by discussion. Once coded, investigators engaged in group work consensus-building meetings to finalise each program theory [65]. Of the 56 interview transcripts, 11 were coded into the audit and feedback program theory, as they included direct reference to the audit and feedback strategy whereas the remaining 45 focussed on other strategies to be published separately (Table 3).

**Table 3:** Demographics of participants coded into the audit and feedback program theory

Item	Participant *n* (%)*
Age group:	
31–45 years	2 (29%)
46–60 years	5 (71%)
Gender:	
Female	6 (86%)
Male	1 (14%)
Years in role:	
1–5 years	4 (57%)
6–10 years	2 (29%)
More than 10 years	1 (14%)

^*^Demographic survey received from seven of the 11 participants

#### Stage 3: translating into generalisable theoretical models for implementation

The program theories were presented to three healthcare quality expert panels for validation. A group of 19 Australian healthcare quality experts was assembled in a breakout meeting during the International Society for Quality in Health Care (ISQua) International Conference in Brisbane, Australia in 2022. A recommended model for the implementation of audit and feedback to reduce unwarranted clinical variation was proposed to the group, and questions were asked to seek confirmation, disconfirmation, and other interpretations (see Table 4 for example). Field notes were taken and incorporated with cross-case comparisons to determine how the same mechanism might produce different outcomes depending on the contextual circumstances. This assisted the research team to uncover potentially generative causal patterns (e.g., conditional causality) for the audit and feedback strategy and regularities and patterns between the proposed mechanisms and outcomes in certain contexts. A similar process was carried out when presenting the audit and feedback program theory to NSW Ministry of Health (*n* = 21) and Agency for Clinical Innovation staff (*n* = 11) to translate into generalisable theoretical models for implementation. These validation groups included some of the original interview participants from stages 1 and 2, providing the opportunity for re-engagement and further feedback from key stakeholders with deep insights into the program of interest, which is an important aspect of realist methods.

**Table 4:** Stage 3 feedback for an implementation model

Recommended model for audit and feedback	Example feedback provided
Make audit design a collaboration between clinicians at diverse hospitals (to ensure local constraints are factored in) and external agencies (to give objective “big picture” input and standardisation across hospitals) to increase acceptance and ownership of audit measures for each site. Clearly communicate the purpose of each measure	One key challenge for clinicians is when audit and feedback is an addition to current workloads rather than a part of practice. We can capture information well in routine workflows. However, we can’t necessarily capture knowledge, as this is qualitative. Scientific rigor of audits is important to ensure sample size and questions are right; otherwise, it can stand in the way of improvement efforts. Clinicians dismiss based on small sample sizes and when they aren’t involved. If the feedback is from an external source, it can be dismissed by clinicians. If clinicians don’t like the audit process, then they can disengage, even if the information might be potentially useful

## Results

Stage 1 led to a description of the initial audit and feedback strategy utilised in the LBVC program. The audit questions and indicators were developed by the Agency for Clinical Innovation from a review of the academic and grey literature related to each of the three conditions, assisted by a clinical expert reference group. This process did not necessarily always utilise the existing state-wide clinical networks. Criteria for feasibility, practicality, and the value of each audit indicator were considered in relation to the burden it might place on hospital clinicians. The indicators then underwent several rounds of refinement both internal and external to the Agency for Clinical Innovation before finalisation. Audits were then pilot-tested prior to their use. An audit team visited each participating hospital, which included two people from the Agency for Clinical Innovation (with variable clinical experience of the conditions) and two people nominated from the local hospital. Retrospective auditing of 40 case medical records took place without necessarily standardising the timeframe of case hospital admissions or whether cases were randomly or purposively sampled. Audit data were returned to the Agency for Clinical Innovation for data cleaning and visualisation prior to a feedback session at the hospital, typically within four to six weeks. Feedback was not always delivered by someone with a clinical background. Hospital staff at the feedback meeting were asked to identify three components of care that were being performed well and three components that could be improved. Unspecified ongoing support was offered by the Agency for Clinical Innovation for local hospital improvement efforts and follow-up audits were organised for hospitals on a voluntary basis. The audit and feedback strategy was refined over time to reduce the number of indicators and data points, increase the involvement of clinical staff or people with a clinical background, and prepare hospitals for the feedback sessions, and provide opportunities for reflection post-feedback.

The audit and feedback implementation strategy is hypothesised to operate through eight mechanisms under different contextual circumstances, underpinned by regular measurement and feedback of both clinical processes and outcomes. The strategy worked well when clinicians (1) felt ownership and buy-in to the process, (2) could make sense of the information provided, (3) were motivated by social influence, and (4) accepted responsibility and accountability for the proposed changes. The success of the strategy was constrained by (5) rationalisation of the status quo, (6) perceptions of unfairness and concerns about data integrity, (7) development of tokenistic improvements plans, and (8) perceived threats to professional autonomy. We report the manifestation of each audit and feedback mechanism to pinpoint key factors that determine success or where the strategy might falter (Table 5). Example quotes used to refute, refine, or confirm the audit and feedback program theory are provided in Table 6.

**Table 5:** Context-mechanism-outcome configuration for the audit and feedback program theory

Context	Mechanism	Outcome
1. Ownership and buy-in		
Audits conducted by an external party but in partnership with local clinicians, to ensure staff have input into the process	Triggers a sense of ownership and buy-in, as clinicians recognise that the audit represents best practice	Trust in the process and capability developed for future audits conducted locally
2. Sensemaking of information feedback		
Local hospitals with leaders who promote a learning culture, open a conduit for clinicians to engage with the auditors and lead the development of improvement plans	Local clinicians are open to hearing about their performance against measures, and on reflection can integrate this information with local, codified knowledge and evidence by proxy, to make sense of the implications for those receiving care	Evidence for implementing changes is provided to clinicians to support their case for local site improvements and educational requirements
3. Motivation and social influence		
Provision of data from an external source that allows comparison and benchmarking across comparable hospitals	Facilitation from an external incentive, peer competition, or credible source	Can overcome external locus of control and trigger motivation to improve or maintain performance
4. Responsibility and accountability		
Repeated feedback and education provided at the point of care to passionate clinicians who can influence practice change	Clinicians assume responsibility for audited components of care	Audit and feedback become an ongoing process and is leveraged to gain managerial support for improvement activities
5. Rationalisation of the status quo		
Perceived lack of partnership in audit process: large number of audit variables used, wrong cohorts audited, unclear, conflicting or absent evidence for audit measures, system barriers to care delivery outside of clinicians’ control. Measures lack meaning and accuracy are considered an impost	Staff’s trust in the results is undermined and tend to focus on rationalising the status quo instead	Disengage from the process and pursue their own priorities from other means of performance measurement and existing practices
6. Perceptions of unfairness and concerns about data integrity		
Audits do not capture local workflows and/or system barriers and/or the uniqueness of local settings	Clinicians perceive the audit as an unfair and unachievable process that sets them up to fail	Focus on defending current practice rather than where things could be improved
Immature communication systems between executive and frontline staff for managing expectations and understanding of the implementation support agency’s role (clinicians can misinterpret the audit as a performance management process rather than a learning opportunity)		
7. Improvement plans that are not followed		
One-off feedback delivered by an external Agency without sufficient time provided for clinicians to digest the information before making improvement decisions or a specific outline of support that could be provided by the external Agency	Feedback does not provide a meaningful foundation for quality improvement	Local hospitals continue working on their own improvement priorities
8. Perceptions of threats to professional autonomy		
Rigid criteria used for audit rather than broad principles of care that are not localised to the local hospital audience	Clinical leaders perceive feedback as a directive and are frustrated that their expertise is not respected	Feedback and proposed changes are resisted or not engaged with because clinicians feel like measures do not adequately capture their work
Audit and feedback delivered to medics by non-medical professional (e.g. community nurse, project officer)

**Table 6:** Example quotes used to refute, refine, or confirm the audit and feedback program theory

Context	Mechanism	Outcome
1. Ownership and buy-in		
I01 Implementer, CHF and diabetes, rural hospital: “It was good to have the ACI team come but also had some local knowledge…I knew the local processes and I knew also, when to chime in and say, oh by the way there's no GP in this town… So, having that local knowledge, definitely did help. Because otherwise I would have been blind to, where to that where to find that information.”	I02 Implementer, ACI: “… the rest were really accepting of the data that we presented. Because there were some of them were actually involved in the development of the audits… so if we went out to the sites where those clinicians were working, we had quite good buy in there.”	103 Implementer, Ministry of Health: “When we went back to the executive group and said here's the data. This is what we're seeing…And I think…that was a bit of a wakeup call in terms of…the compliance that they needed to start working towards. Even though it was a paper chart, they needed to start completing it. So, I think then they had a…diabetes CNC, who then really drove a strong piece of work around compliance on it and educating the nursing staff on it. She did a bit of a improvement project just on the chart there alone. And then conducted her own audits and spot-checked files, whenever she walked into someone's room and started doing stuff like that.”
2. Sensemaking of information feedback		
I04 Implementer, ACI: “[rural hospital] were amazing. We did a lot of sites in [rural hospital]…they were always really keen to hear and reflect and to take it on board and even if they didn't necessarily agree with it…maybe we need to look into that. And they…had that real…learning culture that they wanted to get something out of the audit process.”	I05 Implementer, ACI: “…that sort of turned the tables a little bit in that we could go there. Knowing often what the local context was, if not knowing some of the clinicians really well, and saying hey you guys didn't do so well with this, like say for instance, chest X ray. However, we do know that you're a site that doesn't have X ray coverage from 8 pm at night to late in the morning. So it's quite understandable that you've got a delay that might not be something that you can do something about, nor is that something we should jump up and down about given that you're quite good with the rest of your clinical diagnostic so…we can work with the clinicians to identify okay like it might be identified as unwarranted clinical variation, but is it an excellence based approach versus a perfectionist based approach, I guess, is kind of where that turned the table a little bit…”	I01 Implementer, CHF and diabetes, rural hospital: “Yeah, that was really beneficial, and we use those numbers in the brief, and also the money it was going to save the hospital and patient time transport…so that was really beneficial…”
3. Motivation and social influence		
I07 Clinician, rural hospital, COPD: “Because Ministry of Health is telling the other side…this is our expectations. This is the benchmark we've set this quarter; this is the benchmark, we want you to make, they audited everyone the same, which I think was really important…but at least we're all getting the same audit done to us. So, that’s handy because it means that everyone's been treated the same…”	I04 Implementer, ACI: “Having outcomes is really important and that's one thing I've really learnt. Being on the other side of the bed, is that how important data is and how it can really give some good evidence and some good weights to change. I think there's also a real, depending on the personality, but the scrutiny by peers and what peers are doing, also gives the potential rise to change in practice and improving can improve in practices.”	I07 Clinician, rural hospital, COPD: “So that was a good tool for change like you had managers…sort of executives that couldn’t avoid…the fact that it was on the audit which is why it came back.”
4. Responsibility and accountability		
I02 Implementer, ACI: “…part of our role was to ensure that those correct stakeholders including managers and clinicians were attending…We looked at availability and ensured that they were all in the same room together…for example…one of the rural sites, I think they had two or three clinicians, but they were the right clinicians in the room. So that was part of our planning to ensure they were there.”	I02 Implementer, ACI: “they did have accountability and were taking responsibility for the data, I think, where the gaps were was, they're doing this on top of their work like any other project. We're dedicated to it so, we're doing our best to keep them engaged, and we did a bit of the work but at the end of the day, they do own the project.”	I05 Implementer, ACI: “Yes, some people did use it as a bit of an enabler to support a project or something that they wanted to work on like [rural hospital] has reported to us that they've used LBVC to deliver on some improvements in the service they provide because they provide services to CHF and COPD.”
5. Rationalisation of the status quo		
I06 Clinician, COPD, metro hospital: “it was more about, from my perspective, not having the confidence that we would make change from the audit rather than…not having the confidence in the audit process.”	I07 Clinician, COPD, rural hospital: “…there was a little bit, from the doctors feeling that they questioned how accurate it was… So, I think initially then trusting results is a bit difficult or feeling a bit confused about results. I think it put everyone a little bit on a back foot. When we initially got it, we didn't know this was coming, we didn't understand what you were doing, and they think that they’ve just handed us results and you want us to do something about it. That's what everyone else on a bit of a back foot for us.”	I08 Clinician, rural hospital: “I appreciate it wasn't gaps around the clinicians’ performance, often I was looking around system gaps in terms of the organizational resourcing. And those things are typically beyond the remit of the consultants to address those. And so therefore, I think that's where that level of cynicism grew. I felt that those the system gaps were being pointed at them.”
6. Perceptions of unfairness and concerns about data integrity		
I01 Implementer, CHF and diabetes, rural hospital: “They don't know who their exec. department are now, no one's a real, or who are you talking about. So there seems to be this communication level, and you got your talk about some of our senior clinicians on the floor didn’t know who you're talking about…then there was that not distrust, but that disgruntled…’oh well they don’t that care about us anyway because we never see them’, you know, so that's where I think there's a disconnect. They want us to do this, yet we don't know who you are. And you'd haven’t got a relationship with us. So that relationship was missing…from the managers’ perspective they said well, we don't want to be micromanagers, we want to trust that a clinician will do the right thing and so it's a fine line between how much do you micromanage. And how much do you trust that they're just doing it.”	I05 Implementer, ACI: “Clinicians know exactly what, what it is but the system sometimes provides huge barriers to delivering that. So the focus was never about either can we change a system or how can we work around it. It was always about this the care in doing it. So it's kind of a bit of a setup to fail scenario.”	I04 Implementer, ACI: “So it might be that they weren't documented as being offered smoking cessation support, because the staff knew that they'd done that 10 times before and it wasn't it wasn't going to happen again. So therefore, it wasn't documented, even though they were in the week prior…And the problem with a medical record is it cuts off when the patient is discharged. It doesn't necessarily capture the fact that a phone call was made a week later, or referrals and pulmonary rehab was made a month later, our specific questions were related to…a referral to pulmonary rehab made at that admission. And if it wasn't documented, it didn't happen. But in reality, a lot of times those patients were referred to pulmonary rehab. It just happened a week or two later. Is that acceptable practice? Potentially not, but that came down to the workings of the way that they conducted their service.”
7. Improvement plans that are not followed		
I05 Implementer, ACI: “At the time of the audit…there were a lot of variables and the process was…there would be analysis, and then a report would be sent to the site and feedback would be obtained. And then that data was presented to the broader team of clinicians, some of who may or may not have been aware of the auditing process, and then straight away they were asked to prioritize what activities they were going to do differently… But just asking people straight away there in the room to make a decision on what they're going to prioritize. I think was quite difficult. I don't think was particularly useful.”	I07 Clinician, COPD, rural hospital: “Unfortunately, I think the biggest thing would be having like an audit team, someone that could sit there and say no, that is a player that, when I'm looking at that person is supposed to be with this team. That's the only way that I could see that maybe for hospitals such as mine, make that a little less impacting. Like, they tried to, they've got someone local, but that person probably wasn't the right person.”	I04 Implementer, ACI: “The feeling was our [state government] election [was] soon and LBVC will just be replaced by something else I was actually surprised that it wasn't. But and you know we had a change in Minister as well so there was also that that thinking behind it. You know, I've only been on the management side of health since, 2017, but for those that have been in the system a lot longer. They’re used to…that sort of coming and going out of flavour so if there was that that sense that all the funding is only for a year. There's an election and it's probably going to be something else.”
8. Perceptions of threats to professional autonomy		
I06 Clinician, COPD, metro hospital: “I think that it was to do more with the hard matrix of it. It not being understood in our area, or our situation, our environment, our…understanding the medical team’s reasons for why they variate, why there is the variation. I don’t think that's understood why there is clinical variation. We know it exists, but why does it exist I just don't think that that is understood as much…You know, sometimes you've got to make it work for the patient…if your shoelace breaks you might use a string, but maybe that's not understood.”	I08 Clinician, rural hospital: “There wasn't agreement with it, I suppose. Insulted, I mean, for me, insulted is a stronger term. Just thinking about the various feedbacks that I brought into. It may be but it also instantly led to that frustration because this audit that was done removed from them about the care that they provide, with no understanding. So, I suppose I’d lean more towards the frustration and then that automatically prep the barriers to saying this isn’t going to be relevant for us because they're not measuring us on our on the care that we provide.”	I07 Clinician, rural hospital, COPD: “I would say a lot of that resistance, came from feeling like we were…caught unawares, by what was going on and not understanding to achieve. You felt very put on the spot. We were sat in a room, we sat through a PowerPoint presentation, they go okay here’s how you’re doing. And then, for a period of time feeling like I had no, no feedback or follow up that that I think definitely put my physician that I work with on the backfoot.”

*ACI* Agency for Clinical Innovation, *GP* general practitioner, *CNC* clinical nurse specialist

### Ownership and buy in

Ownership and buy-in underpin the acceptability of the audit and feedback process. Ownership of or buying into the audit indicators and processes was described across multiple data sources as a key driver of practice change. Audits had to adequately capture local workflows in order for clinicians to make meaning of the audit data and buy-in to the process. Local workflows were better captured when audits were conducted by the Agency for Clinical Innovation with the participation of respected local hospital clinicians, such as senior medical, nursing, or allied health. Measures that held local meaning were regarded highly by clinicians. Audits conducted by an external institution remained productive where no clinician reluctance to engage had already taken hold.

### Sensemaking of information feedback

Audits need to make sense to clinicians to be successful. This is about more than understanding the indicators, but also encompasses the ability to incorporate audit data with existing and alternate sources of information. Clinicians engaged well with the audit process when local leaders acted as a conduit between the Agency for Clinical Innovation and the hospitals, by introducing the auditors to key clinical stakeholders. Early and close engagement between the auditors and clinicians created a willingness to receive feedback about their performance against audit indicators, which could then be integrated with localised knowledge to make sense of the implications for those receiving care. Combining the externally validated feedback with local, codified knowledge, supported the development of local business cases that clinicians could use to justify improvement plans to hospital decision-makers.

### Motivation and social influence

Audit and feedback are based on the presumption of motivating individual and collective behaviour change. It was common for clinicians to attribute variation from audit indicators to factors outside their control, in other words, an externalised locus of control. Audit results from other hospitals within the same hospital network opened their eyes to the discrepancy between patient outcomes being achieved compared to elsewhere. This was thought to create an intrinsic motivation to change or maintain performance, especially when the information was considered externally validated and paired with other externally driven incentives.

### Responsibility and accountability

Reducing unwarranted variation in care requires clinicians to take responsibility for the variation and accountability for improvement efforts designed to change practice. Clinicians assumed responsibility for audit indicators when feedback was reinforced at the point of care to passionate and influential colleagues who had the power to effect practice change. Under these circumstances, the audit and feedback cycle was established as an ongoing process and could be used to garner hospital decision-maker support for local improvement plans.

### Rationalisation of the status quo

For audit and feedback to be successful, it needed to overcome the proclivity for clinicians to rationalise the status quo when presented with performance feedback. Audit indicators were considered an impost where they lacked meaning and accuracy. Collecting too many indicators across too diffuse a sample of frustrated and disengaged stakeholders. When decisions on what will be measured come from elsewhere, local hospital staff were less likely to approve and support them. A limited partnership with local hospital clinicians contributed to this perception of too many audit indicators being assessed across the wrong patient cohorts, uncertainty around the evidence to support audit indicators, and selection of indicators where system-level barriers existed beyond clinicians’ control. For example, cynicism was thought to develop when system and organisational resourcing gaps were communicated to medical consultants who, in turn, felt these gaps were beyond their remit to address. Under these circumstances, clinicians tended to dismiss the audits and rationalise the status quo, leading to a general disengagement from the process and pursuit of their own local quality improvement priorities.

### Perceptions of unfairness and concerns about data integrity

Concern about the integrity of the audits and a perception that the process was unfair, or setting clinicians up to fail, permeated some hospitals. Some perceived the variation at their hospital site to be warranted rather than unwarranted, or at least out of their control when considering issues at the level of the organisation or health system that they could not change on the ground. These hospitals were thought to be characterised by audits which did not capture local workflows, recognise system-level barriers, or the uniqueness of local settings and processes. Furthermore, those hospitals with immature systems for communication between executive and frontline staff were unable to manage expectations regarding the Agency for Clinical Innovation’s role in the audit and feedback process. Under these circumstances, clinicians perceived the audit as an unfair and unachievable process that set them up to fail. Results were misinterpreted as a performance management exercise rather than a learning opportunity and led to a defence of current practice instead of focussing on where care could be improved.

### Improvement plans that are not followed

Improvement plans were expected to be developed by the local hospital staff after the audit results were fed back. However, in some cases, these were tokenistic, underdeveloped, or not implemented. Feedback did not provide a meaningful foundation for developing a quality improvement plan when it was delivered by someone external to the organisation from the Agency for Clinical Innovation, especially if they did not have a clinical background. Insufficient time for interpretation of the audit results and an unclear outline of the implementation support being offered by the Agency for Clinical Innovation further constrained the development and implementation of quality improvement plans at a local level, as well as the coordination of efforts at scale to implement changes system-wide. In response, some hospitals continued to focus on alternative quality improvement priorities that did not always align with the aims of the LBVC program.

### Perceptions of threats to professional autonomy

Clinicians operate with a high degree of professional autonomy, which was thought to contribute to their resistance to any rigidity in how the audit indicators were measured and applied. Resistance to change or clinical inertia was apparent when the audit indicators developed by the Agency for Clinical Innovation did not appear to adequately capture local workflows or allow for adaptation. Frustration that clinician expertise was not being respected emphasised this perception of threatened professional autonomy, particularly when feedback was perceived as an external directive to mandate change. These issues manifested when rigid criteria were applied to the measurement of audit indicators rather than considering the indicators as broad principles of care that could be tailored to local settings. Using non-medical clinicians to provide audit feedback to medical staff further reinforced this perceived threat to professional autonomy.

**Fig. 2:**
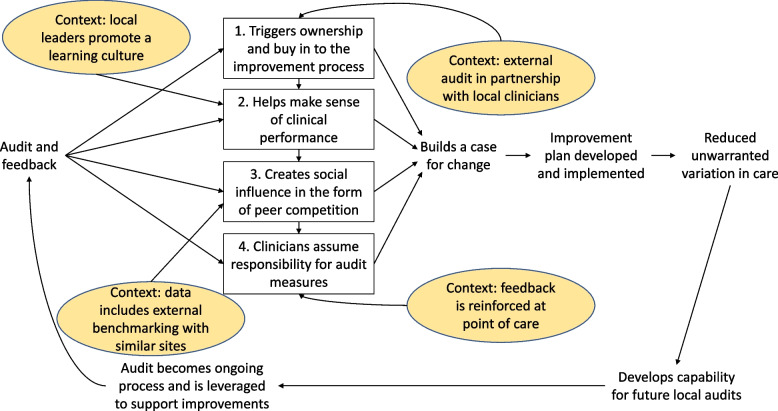
Model for successful audit and feedback implementation at scale

**Fig. 3:**
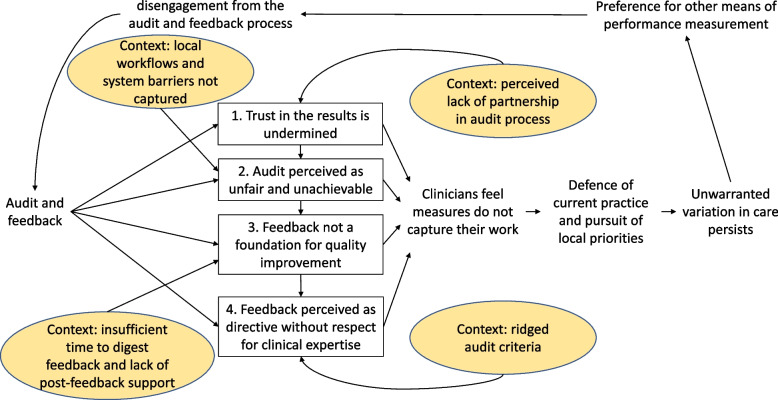
Model for unsuccessful audit and feedback implementation at scale

We propose a Model for Audit and Feedback Implementation at Scale. This model maps the cyclical chain of events underlying the effect on reducing unwarranted clinical variation in inpatient hospital care (Fig. 2) and those underlying unsuccessful reduction of unwarranted clinical variation (Fig. 3). We hypothesise that audit and feedback works through one of four mechanisms (1) triggering ownership and buy in to the improvement process; (2) helping making sense of clinical performance; (3) creating social influence in the form of peer competition; and (4) assuming responsibility for the audit measures. These cyclical and sequential processes are theorised to build a case for change, leading to the development and implementation of improvement plans, which in turn reduce unwarranted variation in care. Contextual influences are represented as yellow ellipses identifying points in the chain of events. Unsuccessful audit and feedback lead to unintended consequences through one of four mechanisms (1) undermining trust in the results; (2) perception of audit as unfair and unachievable; (3) feedback not providing a foundation for quality improvement; and (4) feedback perceived as directive without respect for clinical expertise. These cyclical and sequential processes are theorised to lead clinicians to feel measures do not capture their work, leading to the defence of current practice and pursuit of local priorities, which in turn does not reduce unwarranted variation in care. Contextual influences are represented as yellow ellipses identifying points in the chain of events.

## Discussion

Our study demonstrates that the success of audit and feedback implementation strategies for reducing unwarranted variation in care is contingent upon a variety of contextual circumstances. The program theories describe a series of potential mechanistic causal pathways, which are considered the social responses to audit and feedback. It is these responses that determine the strategy’s success. Different actions taken by various actors can trigger these social responses.

Meaningful partnerships between external auditors and local clinicians appear pivotal in enabling successful audit and feedback cycles. The opportunity for preparation prior to receiving feedback as well as time for post feedback reflection seems to provide a conducive environment for change. Generating sufficient change valence was thought to motivate clinicians, e.g. the more clinicians value the change, the more resolve they will feel to implement the change. Respecting and working in collaboration with clinicians’ professional duty to improve care for their patients more often saw local support for changes in line with the LBVC program. However, there were no magic bullets. The mechanistic causal pathways identified were conditional on various contextual factors. In other words, the causal link between audit and feedback and reduced variation occurs via an underlying generative process existing at certain points in time and space [66]. These casual associations are produced by mechanistic processes, which are themselves important to study.

Implementing an audit and feedback strategy at scale, across an entire health system, is materially different to scaling up a strategy that has already been implemented in other hospital sites. Concurrently delivering an audit and feedback strategy to change processes and systems of care across multiple hospitals requires a macro-view of implementation, by coordinating efforts so that the outcome produced is more than the sum of its parts. This requires a degree of flexibility in how strategies are delivered to balance both fidelity to the intended intervention and adaptation to local contextual needs. Motivating clinicians by comparing their audit results with similar peer hospitals can be achieved by including normative measures for comparison within written reports [67]. For example, in a large audit and feedback trial, Weissman et al. reported that audit recipients that were provided with comparisons to the top 10% of peers improved processes of care more than those who were only compared to the median peer performance [68]. However, feedback needs to describe both desirable and achievable processes and behavioural changes [69], as recipients must believe they have control over that behaviour and commit to change to avoid the risk of recipients rationalising the status quo [70]. Furthermore, our findings reinforce that the amount of feedback in terms of change targets and peer comparisons should be limited to avoid excessive cognitive load and mental effort required to process the feedback, so recipients can more easily make sense of the information provided [71]. The audit and feedback strategy implemented within the LBVC program was not initially designed in a way that could easily enable clinicians to compare audit results data between hospitals from different hospital networks because the sampling method and timeframe was not standardised. This made it difficult for implementors to coordinate efforts at scale across the state-wide health system.

Providing feedback on components of care that align with the existing goals and priorities of individual clinicians and organisations could be achieved by conducting a local needs assessment before providing feedback. That local needs assessment might facilitate the engagement and attention of recipients and maintain their self-efficacy and control of the process [71]. However, this does not mean that clinician values and norms cannot be challenged. Foy et al. previously demonstrated that clinical practice recommendations which were considered incompatible with clinician values and norms can produce greater behaviour changes than those considered compatible [72]. This is likely due to the greater potential for improvements and the likelihood that there might only be limited room for improvement for recommendations already compatible with clinician norms.

Our findings that local leaders could provide a beneficial conduit between the external auditor and hospitals aligns with previous research supporting feedback as more effective when delivered by senior colleagues [73]. Social pressures have been shown to influence intentions to change clinical practice when driven by respected local hospital clinicians [73]. In circumstances without the brokerage of local clinical leaders, it is possible that the feedback could have been perceived as an external directive to mandate change. Feedback is considered less effective when delivered by a regulatory agency, as it can be construed as punitive rather than supportive [69, 74]. Therefore, extra efforts might need to be made by external audit and feedback providers to ensure psychological safety, target behaviours considered important to the recipient, and provide reassuring messages to avoid invoking a defensive reaction to the feedback [71].

Our study findings broadly align with the “high-confidence hypotheses” put forward by Brown et al.’s Clinical Performance Feedback Intervention Theory (CP-FIT) [32]. However, we have identified where these findings can be used to refine this existing theory and contribute to the rich agenda focusing on how to optimise the effects of audit and feedback interventions. Our audit and feedback program theories for large-scale system change challenge three of the CP-FIT hypotheses: specificity, exclusions, and delivery to a group. The specificity hypothesis posits that feedback should be provided based on individual health professional performance rather than at the team or organisation level [75]. Our study identified mechanisms by which audit and feedback could be provided in group settings and produce hospital-wide quality improvement plans. Changing systems of care requires a focus on multilevel, multifactorial, and multidimensional processes that often are beyond the level of an individual clinician. This also appears to contradict the delivery to a group hypothesis that feedback should be delivered to groups of recipients [76]. We posit that feedback should be provided at the level where the behaviour change is required to achieve the desired outcome (e.g. individual, team, organisational in combination). Levesque and Sutherland previously outlined eight levers for change enabled by performance information that account for these different levels of the system where behaviour change is desired [77], and provide a potential framework for integrating future audit and feedback strategies with large system value-based healthcare programs. According to our findings, the exclusions hypothesis that recipients be allowed to exclude patients they consider inappropriate from the audit and feedback process [78] potentially risks gaming of the audit and feedback process. In our study, retrospective audits of medical records without standardising the timeframe of hospital admissions or sampling approaches reportedly led to high levels of heterogeneity in the cases selected and limited the ability to support state-wide implementation at scale by comparing audit results across hospitals. We posit that a standardised timeframe and sampling approach be used to ensure audits most closely reflect real-world clinical practice and ensure external generalisability of audit results.

### Strengths and limitations

Our realist study integrated context within hypothesised causal models. In developing theory for implementation strategies in healthcare, it is not uncommon to divide contextual factors, theoretical mechanisms, and implementation outcomes, and study these factors in isolation [79, 80]. This can lead to competing theories of change in different contextual circumstances [54]. However, it is more likely that they are alternate theories of change which could occur in any setting depending on interactions with these contextual factors. This concept of latent or unrealised mechanisms in realist research paradigms allowed the elucidation of “dormant” change mechanisms which could be triggered by certain contextual circumstances [81].

The three LBVC initiatives targeting inpatient variation in care were complex interventions that included more than just audit and feedback. It is possible that our focus on the audit and feedback strategy within these initiatives did not sufficiently capture complex interactions between the audit and feedback strategy and other approaches, such as defining best practice management, capability building to support best practice management, and systematising local processes to detect and address unwarranted clinical variation. The research team was not involved in developing or delivering the audit and feedback strategy, limiting our ability to report the strategy according to the AACTT framework [82]. The strategy was also modified between sites and over time, further limiting our ability to provide specific descriptions according to the AACTT framework. Despite a large amount of qualitative data collected, relatively few interview participants provided information that could be coded to the audit and feedback program theory. However, realist hypotheses are not reliant on theoretical saturation obtained in a pre-specified number of qualitative interviews but through a focus on relevance, credibility, and rigour [57]. Furthermore, the limited availability of quantitative data outlined in our study protocol constrained the ability to triangulate qualitative and quantitative data to provide a more robust program theory for audit and feedback in large-system value-based healthcare initiatives.

## Conclusion

Our realist study identified eight mechanisms by which audit and feedback implementation strategies may impact unwarranted clinical variation in hospital care. Ownership and buy-in were more likely to be achieved when audits were conducted in collaboration with respected local hospital clinicians to better capture local workflows. Furthermore, where these local leaders facilitated engagement between the external auditors and local clinicians, performance on audit indicators was better able to be integrated with localised knowledge to make sense of the practice change implications. Comparisons with peer equivalent hospitals motivated a sense of positive social influence if clinicians considered the audit indicators and processes of care to be within their responsibility and locus of control. Where there was no meaningful partnership between auditors and local clinicians, too many audit indicators were assessed that lacked the necessary meaning and accuracy to be actionable, leading to clinicians rationalising existing care processes rather than focussing on potential areas for quality improvement. This lack of partnership can create a perception of unfairness when local workflows are not adequately accounted for in the audits. Improvement plans were underdeveloped or not implemented when sufficient time to interpret audit results and a clear indication of the level of ongoing implementation support was not provided. Finally, where clinical expertise was not respected because of rigid audit indicator criteria, the process was considered a threat by some to professional autonomy.

Recommendations for future audit and feedback strategies include limiting the number of audit indicators and data points, ensuring the involvement of clinical staff and local leaders in delivering feedback, preparing hospital staff for the feedback sessions, and providing opportunities for reflection post-feedback. Future research could determine how to optimise benefits and avoid unintended outcomes by focussing on head-to-head comparisons of audit and feedback strategies, incorporating these elements with embedded process evaluations to determine the interaction effect of the hypothesised contextual factors in our program theories.

### Supplementary Information

**Additional file 1.** Audit and feedback program logic for CHF and COPD initiatives.

**Additional file 2.** Audit and feedback program logic for Inpatient Management of Diabetes Mellitus Leading Better Value Care initiative.

**Additional file 3.** RAMESES II reporting standards for realist evaluations.

**Additional file 4.** Study protocol adaptations.

**Additional file 5.** Initial audit and feedback program theory for CHF, COPD, and diabetes initiatives.

## Data Availability

The datasets generated and analysed during the current study are not publicly available due to potential concerns regarding the identifiability of participants but may be available from the corresponding author on reasonable request.

## Abbreviations

NSW: New South Wales
LBVC: Leading Better Value Care
COPD: Chronic obstructive pulmonary disease
CHF: Chronic heart failure
BGL: Blood glucose level
ACI: Agency for Clinical Innovation
GP: General practitioner
CNC: Clinical nurse specialist

## References

[1] Morgan S, Cunningham C, Hanley G, Mooney D. The British Columbia medical and hospital atlas: a companion to the British Columbia Rx atlas, 2nd edition. British Columbia: Centre for Health Services and Policy Research (CHSPR); 2009.

[2] Dartmouth Medical School. The Dartmouth atlas of health care. Chicago: American Hospital Association; 1996.

[3] McGlynn EA, Asch SM, Adams J, Keesey J, Hicks J, DeCristofaro A (2003). The quality of health care delivered to adults in the United States. N Engl J Med.

[4] Runciman WB, Hunt TD, Hannaford NA, Hibbert PD, Westbrook JI, Coiera EW (2012). CareTrack: assessing the appropriateness of health care delivery in Australia. Med J Aust.

[5] Breton ER, Fuemmeler BF, Abroms LC (2011). Weight loss-there is an app for that! But does it adhere to evidence-informed practices?. Transl Behav Med.

[6] Haines TP, Bowles K-A, Mitchell D, O'Brien L, Markham D, Plumb S (2017). Impact of disinvestment from weekend allied health services across acute medical and surgical wards: 2 stepped-wedge cluster randomised controlled trials. PLoS Med.

[7] Kelly P, Baker G, McAdam C, Milton K, Richards J, Foster C (2016). Critique of 'The physical activity myth' paper: discussion of flawed logic and inappropriate use of evidence. Br J Sports Med.

[8] Saini V, Brownlee S, Elshaug AG, Glasziou P, Heath I (2017). Addressing overuse and underuse around the world. Lancet.

[9] Sarkies MN, Bowles KA, Skinner EH, Haas R, Mitchell D, O'Brien L (2016). Do daily ward interviews improve measurement of hospital quality and safety indicators? A prospective observational study. J Eval Clin Pract.

[10] Sutherland K, Levesque J-F. Unwarranted clinical variation in health care: definitions and proposal of an analytic framework. J Eval Clin Pract. 2019;26. 10.1111/jep.13181. 10.1111/jep.13181 PMC7317701 31136047

[11] Wennberg JE (2002). Unwarranted variations in healthcare delivery: implications for academic medical centres. BMJ.

[12] Birkmeyer JD, Reames BN, McCulloch P, Carr AJ, Campbell WB, Wennberg JE (2013). Understanding of regional variation in the use of surgery. Lancet.

[13] Lutfey KE, Link CL, Marceau LD, Grant RW, Adams A, Arber S (2009). Diagnostic certainty as a source of medical practice variation in coronary heart disease: results from a cross-national experiment of clinical decision making. Med Decis Making.

[14] Mayer M, Naylor J, Harris I, Badge H, Adie S, Mills K (2017). Evidence base and practice variation in acute care processes for knee and hip arthroplasty surgeries. PLoS ONE.

[15] Offerhaus PM, Geerts C, de Jonge A, Hukkelhoven CWPM, Twisk JWR, Lagro-Janssen ALM (2015). Variation in referrals to secondary obstetrician-led care among primary midwifery care practices in the Netherlands: a nationwide cohort study. BMC Pregnancy Childbirth.

[16] Wennberg J, Gittelsohn. Small area variations in health care delivery. Science. 1973;182(4117):1102–8. 10.1126/science.182.4117.1102. 10.1126/science.182.4117.1102 4750608

[17] de Jong JD, Westert GP, Lagoe R, Groenewegen PP. Variation in hospital length of stay: do physicians adapt their length of stay decisions to what is usual in the hospital where they work? Health Serv Res. 2006;41(2):374–94. 10.1111/j.1475-6773.2005.00486.x. 10.1111/j.1475-6773.2005.00486.x PMC1702523 16584454

[18] Moen EL, Bynum JP, Austin AM, Skinner JS, Chakraborti G, O'Malley AJ (2018). Assessing variation in implantable cardioverter defibrillator therapy guideline adherence with physician and hospital patient-sharing networks. Med Care.

[19] McAlister FA, Lin M, Bakal J, Dean S (2018). Frequency of low-value care in Alberta, Canada: a retrospective cohort study. BMJ Qual Saf.

[20] Segal JB, Bridges JFP, Chang H-Y, Chang E, Nassery N, Weiner J (2014). Identifying possible indicators of systematic overuse of health care procedures with claims data. Med Care.

[21] Wennberg JE, Freeman JL, Culp WJ (1987). Are hospital services rationed in New Haven or over-utilised in Boston?. Lancet.

[22] Wennberg JE, Freeman JL, Shelton RM, Bubolz TA (1989). Hospital use and mortality among Medicare beneficiaries in Boston and New Haven. N Engl J Med.

[23] Fisher ES, Wennberg JE, Stukel TA, Sharp SM (1994). Hospital readmission rates for cohorts of Medicare beneficiaries in Boston and New Haven. N Engl J Med.

[24] Cutler D, Skinner JS, Stern AD, Wennberg D (2019). Physician beliefs and patient preferences: a new look at regional variation in health care spendingf. Am Econ J Econ Policy.

[25] Elshaug AG, Watt AM, Mundy L, Willis CD (2012). Over 150 potentially low-value health care practices: an Australian study. Med J Aust.

[26] Saini V, Garcia-Armesto S, Klemperer D, Paris V, Elshaug AG, Brownlee S (2017). Drivers of poor medical care. Lancet.

[27] Braithwaite J, Glasziou P, Westbrook J (2020). The three numbers you need to know about healthcare: the 60–30-10 Challenge. BMC Med.

[28] Gauld R, Horwitt J, Williams S, Cohen AB (2011). What Strategies Do US Hospitals Employ to Reduce Unwarranted Clinical Practice Variations?. Am J Med Qual.

[29] Ivers N, Jamtvedt G, Flottorp S, Young JM, Odgaard-Jensen J, French SD, et al. Audit and feedback: effects on professional practice and healthcare outcomes. Cochrane Database Syst Rev. 2012(6):CD000259. 10.1002/14651858.CD000259.pub3. 10.1002/14651858.CD000259.pub3 PMC11338587 22696318

[30] Colquhoun HL, Brehaut JC, Sales A, Ivers N, Grimshaw J, Michie S (2013). A systematic review of the use of theory in randomized controlled trials of audit and feedback. Implement Sci.

[31] Sales A, Smith J, Curran G, Kochevar L. Models, strategies, and tools. Theory in implementing evidence-based findings into health care practice. J Gen Intern Med. 2006;21 Suppl 2(S2):S43–9. 10.1111/j.1525-1497.2006.00362.x. 10.1111/j.1525-1497.2006.00362.x PMC2557135 16637960

[32] Brown B, Gude WT, Blakeman T, van der Veer SN, Ivers N, Francis JJ (2019). Clinical Performance Feedback Intervention Theory (CP-FIT): a new theory for designing, implementing, and evaluating feedback in health care based on a systematic review and meta-synthesis of qualitative research. Implement Sci.

[33] Morrell C, Harvey G, Kitson A (1997). Practitioner based quality improvement: a review of the Royal College of Nursing's dynamic standard setting system. Qual Health Care.

[34] Grant AM, Guthrie B, Dreischulte T (2014). Developing a complex intervention to improve prescribing safety in primary care: mixed methods feasibility and optimisation pilot study. BMJ Open.

[35] Drummond M, Sculpher MJ, Claxton K, Stoddart GL, Torrance GW. Methods for the economic evaluation of health care programmes. Fourth edition. ed. Oxford; New York: Oxford University Press; 2015. xiii, 445.

[36] Koff E, Lyons N (2020). Implementing value-based health care at scale: the NSW experience. Med J Aust.

[37] Health Economics and Evaluation Team. Reducing unwarrented clinical variation in chronic obstructive pulmonary disease and chronic heart failure: monitoring and evaluation plan. Sydney: NSW Agency for Clinical Innovation; 2017. Available from: https://aci.health.nsw.gov.au/statewide-programs/lbvc/chronic-heart-failure.

[38] Health Economics and Evaluation Team. Inpatient Management of Diabetes Mellitus: Monitoring and evaluation plan. Australia: NSW Agency for Clinical Innovation; 2017. Available from: https://aci.health.nsw.gov.au/statewide-programs/lbvc/inpatient-management-of-diabetes-mellitus.

[39] de Souza DE (2013). Elaborating the Context-Mechanism-Outcome configuration (CMOc) in realist evaluation: A critical realist perspective. Evaluation.

[40] Lewis CC, Boyd MR, Walsh-Bailey C, Lyon AR, Beidas R, Mittman B (2020). A systematic review of empirical studies examining mechanisms of implementation in health. Implement Sci.

[41] Pawson R (2006). Evidence-based policy.

[42] Pawson R, Tilley N (1997). Realistic evaluation.

[43] Funnell S, Rogers P (2011). Purposeful program theory: effective use of theories of change and logic models.

[44] Jagosh J (2020). Retroductive theorizing in Pawson and Tilley's applied scientific realism. J Crit Realism.

[45] Sarkies M, Long J, Pomare C, Wu W, Clay-Williams R, Nguyen H, et al. Avoiding unnecessary hospitalisation for patients with chronic conditions: a systematic review of implementation determinants for hospital avoidance programmes. Implement Sci. 2020;15. 10.1186/s13012-020-01049-0. 10.1186/s13012-020-01049-0 PMC7579904 33087147

[46] Jackson S, Kolla G (2012). A new realistic evaluation analysis method: linked coding of context, mechanism and outcome relationships. Am J Eval.

[47] Wong G, Pawson R, Owen L (2011). Policy guidance on threats to legislative interventions in public health: A realist synthesis. BMC Public Health.

[48] Lacouture A, Breton E, Guichard A, Ridde V. The concept of mechanism from a realist approach: A scoping review to facilitate its operationalization in public health program evaluation. Implement Sci. 2015;10. 10.1186/s13012-015-0345-7. 10.1186/s13012-015-0345-7 PMC4628377 26519291

[49] Sarkies M, Robinson S, Ludwick T, Braithwaite J, Nilsen P, Aarons G, et al. Understanding implementation science from the standpoint of health organisation and management: an interdisciplinary exploration of selected theories, models and frameworks. J Health Organ Manag. 2021. 10.1108/JHOM-02-2021-0056.

[50] Braithwaite J, Churruca K, Long J, Ellis L, Herkes J. When complexity science meets implementation science: A theoretical and empirical analysis of systems change. BMC Med. 2018;16. 10.1186/s12916-018-1057-z. 10.1186/s12916-018-1057-z PMC5925847 29706132

[51] Sarkies M, Francis-Auton E, Long J, Partington A, Pomare C, Nguyen H (2020). Implementing large-system, value-based healthcare initiatives: A realist study protocol for seven natural experiments. BMJ Open.

[52] Wong G, Westhorp G, Manzano A, Greenhalgh J, Jagosh J, Greenhalgh T (2016). RAMESES II reporting standards for realist evaluations. BMC Med.

[53] Francis-Auton E, Sarkies MN, Pomare C, Long JC, Hardwick R, Nguyen HM (2022). Real Talk: A Realist Dialogic Approach in a Realist Evaluation. Int J Qual Methods.

[54] Sarkies M, Francis-Auton E, Long J, Pomare C, Hardwick R, Braithwaite J. Making implementation science more real. BMC Med Res Methodol. 2022;22. 10.1186/s12874-022-01661-2. 10.1186/s12874-022-01661-2 PMC9233332 35752754

[55] Bureau of Health Information. New South Wales and the COVID-19 pandemic from 2020 to 2022. Sydney: BHI; 2023. Available from: https://www.bhi.nsw.gov.au/BHI_reports/healthcare_in_focus/new_south_wales_and_the_covid-19_pandemic_from_2020_to_2022.

[56] Long JC, Sarkies MN, Auton EF, Nguyen HM, Pomare C, Hardwick R (2022). Conceptualising contexts, mechanisms and outcomes for implementing large-scale, multisite hospital improvement initiatives: a realist synthesis. BMJ Open.

[57] Manzano A. The craft of interviewing in realist evaluation. Evaluation. 2016;22. 10.1177/1356389016638615.

[58] Mukumbang F, Marchal B, Van Belle S, Van Wyk B (2019). Using the realist interview approach to maintain theoretical awareness in realist studies. Qual Res.

[59] Mukumbang FC, Marchal B, Van Belle S, van Wyk B (2020). Using the realist interview approach to maintain theoretical awareness in realist studies. Qual Res.

[60] Gilmore B, McAuliffe E, Power J, Vallières F (2019). Data analysis and synthesis within a realist evaluation: toward more transparent methodological approaches. Int J Qual Methods.

[61] Hewitt G, Sims S, Harris R (2012). The realist approach to evaluation research: an introduction. Int J Ther Rehabil.

[62] Mark M, Henry G, Julnes G (2004). A realist theory of evaluation practice. N Dir Eval.

[63] Salter KL, Kothari A (2014). Using realist evaluation to open the black box of knowledge translation: a state-of-the-art review. Implement Sci.

[64] Rogers P. Program Theory: Not Whether Programs Work but How They Work. 2002. 209–32.

[65] De Brún A, McAuliffe E. Identifying the context, mechanisms and outcomes underlying collective leadership in teams: building a realist programme theory. BMC Health Serv Res. 2020;20. 10.1186/s12913-020-05129-1. 10.1186/s12913-020-05129-1 PMC7106698 32228574

[66] Blossfeld HP. Causation as a Generative Process. The Elaboration of an Idea for the Social Sciences and an Application to an Analysis of an Interdependent Dynamic Social System. In: Engelhardt H, Kohler HP, Fürnkranz-Prskawetz A, editors. Causal Analysis in Population Studies. The Springer Series on Demographic Methods and Population Analysis, vol 23. Dordrecht: Springer; 2009. 10.1007/978-1-4020-9967-0_5.

[67] Kiefe CI, Allison JJ, Williams OD, Person SD, Weaver MT, Weissman NW (2001). Improving quality improvement using achievable benchmarks for physician feedback: a randomized controlled trial. JAMA.

[68] Weissman NW, Allison JJ, Kiefe CI, Farmer RM, Weaver MT, Williams OD (1999). Achievable benchmarks of care: the ABCs of benchmarking. J Eval Clin Pract.

[69] Hysong SJ, Best RG, Pugh JA (2006). Audit and feedback and clinical practice guideline adherence: Making feedback actionable. Implement Sci.

[70] Locke EA, Latham GP. Building a practically useful theory of goal setting and task motivation. A 35-year odyssey. Am Psychol. 2002;57(9):705–17. 10.1037//0003-066x.57.9.705. 10.1037//0003-066x.57.9.705 12237980

[71] Colquhoun HL, Carroll K, Eva KW, Grimshaw JM, Ivers N, Michie S (2017). Advancing the literature on designing audit and feedback interventions: identifying theory-informed hypotheses. Implement Sci.

[72] Foy R, MacLennan G, Grimshaw J, Penney G, Campbell M, Grol R (2002). Attributes of clinical recommendations that influence change in practice following audit and feedback. J Clin Epidemiol.

[73] Ajzen I (1991). The theory of planned behavior. Organ Behav Hum Decis Process.

[74] Kluger AN, Van Dijk D (2010). Feedback, the various tasks of the doctor, and the feedforward alternative. Med Educ.

[75] Nessim C, Bensimon CM, Hales B, Laflamme C, Fenech D, Smith A (2012). Surgical site infection prevention: a qualitative analysis of an individualized audit and feedback model. J Am Coll Surg.

[76] Hysong SJ, Knox MK, Haidet P. Examining clinical performance feedback in Patient-Aligned Care Teams. J Gen Intern Med. 2014;29 Suppl 2(Suppl 2):S667–74. 10.1007/s11606-013-2707-7. 10.1007/s11606-013-2707-7 PMC4070233 24715398

[77] Levesque J-F, Sutherland K (2017). What role does performance information play in securing improvement in healthcare? a conceptual framework for levers of change. BMJ Open.

[78] Chadwick LM, MacPhail A, Ibrahim JE, McAuliffe L, Koch S, Wells Y (2016). Senior staff perspectives of a quality indicator program in public sector residential aged care services: a qualitative cross-sectional study in Victoria. Australia Aust Health Rev.

[79] May CR, Johnson M, Finch T (2016). Implementation, context and complexity. Implement Sci.

[80] Wensing M, Grol R (2019). Knowledge translation in health: how implementation science could contribute more. BMC Med.

[81] Jagosh J (2019). Realist synthesis for public health: building an ontologically deep understanding of how programs work, for whom, and in which contexts. Annu Rev Public Health.

[82] Presseau J, McCleary N, Lorencatto F, Patey AM, Grimshaw JM, Francis JJ (2019). Action, actor, context, target, time (AACTT): a framework for specifying behaviour. Implement Sci.

