# Supplementary material for: Audit and feedback to reduce unwarranted clinical variation at scale: a realist study of implementation strategy mechanisms
Source: Implement Sci. 2023 Dec 11;18:71. doi: 10.1186/s13012-023-01324-w (PMC10714549; doi:10.1186/s13012-023-01324-w)
Supplement: Supplementary file 1 — Additional file 1. Audit and feedback program logic for CHF and COPD initiatives. [file 13012_2023_1324_MOESM1_ESM.docx]

**Additional File 1.** Audit and feedback program logic for CHF and COPD initiatives

| **Inputs** | **Activities** | **Short term outcomes** | **Medium term outcomes** | **Long term outcomes** |
| --- | --- | --- | --- | --- |
| Clinical practice guidelines available | ACI recommends measurement tools and standards | Local measurement systems are established | Clinical process measures are collected and monitored | Local hospitals have processes in place to monitor and provide routine feedback on clinical variation |
| Ministry of Health, ACI, and local hospitals partners to deliver program | Clinical practice guidelines disseminated | Local hospital staff are aware of best practice and identify gaps in care provision | Local hospital staff design improvement plans to align routine care with best practice | Local hospital staff have the knowledge, skills and confidence required to improve clinical practice in identified areas and include patients are carers in decision making |
| ACI supports:   - Audit and feedback - Clinician forum - Improvement support - Online collaboration portal - Training on best practice care - Measurement tools - Patient and carer resources | Patient outcome and experience measures are routinely collected | Local hospital staff value patient reported measures to inform care | Local hospital staff provide tailored education and support to meet patient and carers wishes | Local hospital staff link patients and carers with service providers who can help meet their ongoing needs |

*Modified to focus on audit and feedback strategy from Agency for Clinical Innovation Reducing Unwarranted Clinical Variation in Chronic Obstructive Pulmonary Disease and Chronic Heart Failure: Monitoring and Evaluation Framework retrieved 20/04/2023 from: https://aci.health.nsw.gov.au/__data/assets/pdf_file/0006/456423/170629-RUCV-CHF-COPD-M-and-E-plan.pdf
