# Supplementary material for: Audit and feedback to reduce unwarranted clinical variation at scale: a realist study of implementation strategy mechanisms
Source: Implement Sci. 2023 Dec 11;18:71. doi: 10.1186/s13012-023-01324-w (PMC10714549; doi:10.1186/s13012-023-01324-w)
Supplement: Supplementary file 2 — Additional file 2. Audit and feedback program logic for Inpatient Management of Diabetes Mellitus Leading Better Value Care initiative. [file 13012_2023_1324_MOESM2_ESM.docx]

**Additional File 2.** Audit and feedback program logic for Inpatient Management of Diabetes Mellitus Leading Better Value Care initiative

| **Inputs** | **Activities** | **Short term outcomes** | **Medium term outcomes** | **Long term outcomes** |
| --- | --- | --- | --- | --- |
| Evidence check on best practice insulin management and development of audit tools and subcutaneous insulin chart | ACI support defining elements of best practice and developing standardised audit to inform improvements and benchmarking | Local hospitals audit, identify areas for improvement, and develop improvement plans for early identification, risk assessment, referral pathways, and enablement strategies for people with diabetes | Clinical teams have reliable process for handover of care and people with diabetes are involved in shared care decision making | People with diabetes are provided with support for their condition, are confident with insulin administration and that their diabetes is optimally managed |
| Diabetes taskforce provides advice on best practice in-hospital care of diabetes | ACI offers implementation and re-design support | Local hospital staff are aware and utilise capability building resources and support | Local clinical practice improvements are made in in-hospital management of people with diabetes requiring insulin | People with diabetes in hospital are provided with best practice insulin management |
| Patient journey mapping and defining of best practice care elements and Leading Better Value Care program introduced by Ministry of Health | Ministry of Health and ACI provide resources and present case for change to local hospitals | Local hospitals facilitate leadership, workforce and governance structure to support change | Local hospitals monitor diabetes care | Audit systems are in place for sustainability and ongoing improvement |

*Modified to focus on audit and feedback strategy from ACI Health Economics and Evaluation Team Inpatient Management of Diabetes Mellitus: Monitoring and Evaluation Plan retrieved 20/04/2023 from: https://aci.health.nsw.gov.au/__data/assets/pdf_file/0010/373096/170628-Diabetes-ME-plan.pdf
