# Supplementary material for: Audit and feedback to reduce unwarranted clinical variation at scale: a realist study of implementation strategy mechanisms
Source: Implement Sci. 2023 Dec 11;18:71. doi: 10.1186/s13012-023-01324-w (PMC10714549; doi:10.1186/s13012-023-01324-w)
Supplement: Supplementary file 4 — Additional file 4. Study protocol adaptations. [file 13012_2023_1324_MOESM4_ESM.docx]

**Additional File 4.** Study protocol adaptations

Several adaptations were made from our protocol. Analysis of internal documents pertaining to the LBVC program were originally planned to inform the development of initial program theories during Stage 1; instead, they were used to refine and test the program theories during Stage 2 due to delays in approval for access. Less than 30 program implementers and evaluators were interviewed in Stage 1 because we achieved sample saturation earlier than expected. More than 30 local hospital staff were interviewed in Stage 2 because a higher number of key informants were identified than anticipated. We were unable to re-interview key informants due to intermittent lockdowns due to COVID-19 outbreaks and the accompanying increased workload on health system staff. Program evaluation data were not made available to the research team at the level of granularity required to perform quantitative analysis and triangulate with the qualitative data. Deviations from realist study protocols do not necessarily constitute a risk of bias, as every detail of the research question, scope and design cannot always be pre-specified due to the iterative and exploratory nature of these studies, which means that the important factors for examination emerge as program theories develop.
