# Supplementary material for: Audit and feedback to reduce unwarranted clinical variation at scale: a realist study of implementation strategy mechanisms
Source: Implement Sci. 2023 Dec 11;18:71. doi: 10.1186/s13012-023-01324-w (PMC10714549; doi:10.1186/s13012-023-01324-w)
Supplement: Supplementary file 5 — Additional file 5. Initial audit and feedback program theory for CHF, COPD, and diabetes initiatives. [file 13012_2023_1324_MOESM5_ESM.docx]

**Additional File 5.** Initial audit and feedback program theory for CHF, COPD and diabetes initiatives

| **Context** | **Actor** | **Mechanism** | **Theory** | **Outcome** | **INTERVIEW QUESTIONS** |
| --- | --- | --- | --- | --- | --- |
| 1a. In settings where staff have input towards the audit measures and the source/process is trustworthy | 1a. Clinical leaders | 1a. Ownership and buy in:  Triggers a sense of ownership and buy in | 1a. Behaviour change theory (e.g. COM-B, TDF) (1-3) | 1a. Trust in measures:  Trust that process measures accurately represent indicators for best practice | We’ve heard that when people feel they have a say/buy in in developing audit measures, they have more trust in those measures. Has that been the case?   - Why was that? *(prompt for mechanism)* |
| 1b. In settings where the most important measures from the clinicians’ perspective are not captured | 1b. Clinical leaders | 1b. Rationalising:  Diverts attention from improvements and towards rationalising the status quo | 1b. Feedback intervention theory (4), heuristic-systematic model of information processing (5), theory of cognitive dissonance (6), self-affirmation theory (7) | 1b. Measures dismissed:  Clinical leaders confirm and reinforce existing behaviour using pre-existing attitudes and beliefs | Was it like that for everyone?  Is there an example of when that didn’t work?   - Who? - Where? - Why do you think that was the case? |
| 2a. In settings where measures are transparent and consistently used across organisations over time | 2a. Clinical staff | 2a. Sensemaking:  Performance against measures can be integrated with local, codified knowledge and evidence by proxy, to make sense of the implications for those receiving care | 2a. Chain of codified knowledge (8) | 2a. Understanding of change:  Clinicians understand what changes or lack of change in clinical processes signifies for patient care | In your experience, did you think the audit measures were transparent? (C)  And did that help with your understanding of how the initiative benefited patient care? (O)  Why was that the case? (M)  Was it because you were able to integrate [mechanisms box]? |
| 2b. In settings where measurement and feedback systems are not transparent, focus on single time points rather than trends and relative values (differences) are small | 2b. Clinical staff | 2b. Measures considered unfair:  Measures are considered unfair and unachievable due to perceived uniqueness of local setting | 2b. Reference group theory (9), goal setting theory (10), theory of cognitive dissonance (6), self-affirmation theory (7) | 2b. Focus on defending practice:  Focus can become more on perception of practice integrity and moves away from patient experience | Are you aware of places where this hasn’t happened/ (or where it has worked)? |
| 3a. Where clinicians and organisations agree to principles of care (rather than being told what to do) | 3a. Clinical leaders | 3a. Competition generated by social influence:  Competition between organisations/peers encourages action, based on comparative status | 3a. Social comparison theory (11), persuasion theory (12), social norms theory (13), reference group theory (9) | 3a. Motivation:  Motivation to improve or maintain performance | Are you aware of what’s going on at other hospitals in terms of their audit and feedback reports? (M)  Based on your knowledge on what X hospital is doing, have things changed here? How have they changed? What was it like before? And what are the consequences of that? (O)  Was it the case that it also lead to you feeling motivated to improve?  (C) *tbd when refine IPT* |
| 3b. Broad principles of care are not set, and ridged criteria do not allow for local tailoring | 3b. Clinical leaders | 3b. Threat to autonomy:  Clinical leaders perceive feedback as a directive and perceive that their expertise is not respected | 3b. | 3b. Resistance:  Feedback and proposed changes are resisted |  |
| 4a. When feedback is delivered to the right person or group of people who have the power to change practice (including management support) | 4a. Clinical leaders | 4a. Responsibility/accountability:  Clinical leaders assume responsibility for the measures and are willing to accept accountability towards them | 4a. Organisation change readiness theory (14) | 4a. Develop improvement plan:  Clinical and non-clinical leaders develop an improvement plan, based on the audit results | How did the clinical leaders respond to the A&F feedback?  We’ve heard that it’s important that feedback goes to the people with the power to make changes, as it can lead to improvements. Was that the case here? |
| 4b. Feedback presented by someone with an inaccurate understanding of social setting – outsider or someone without authority | 4b. | 4b. Feedback not accepted:  Leaders do not accept or commit to feedback provided by auditor | 4b. Social identity theory (15) | 4b. Tokenistic plan:  Clinical and non-clinical leaders only engage in improvement planning in a superficial, tokenistic basis | *Not clinical leaders*  Could you tell us who did receive the feedback?  Who? How? What?  What was the results of this?  Why do you think that was the case? |
| 5a. Knowing that processes are being monitored providing sufficient incentive to encourage change | 5a. Clinical staff | 5a. Stop and think:  Prompts clinicians to reflect on current practice and ensure it is in line with guidelines | 5a. Behaviour change theory (e.g. COM-B, TDF) (1-3) Foucault and panopticism thinking on surveillance (16) | 5a. Action improvements in care:  Clinical staff will take action to improve care, based on the improvement plan and process measures | If your processes were being monitored, did that have any impact on your performance?  What was the impact that it had?  Why do you think that was the case?  (Did it prompt you to stop and reflect?) |
| 5b. History of change initiative churn in the organisation and processes are being monitored | 5b. | 5b. Perception that initiatives are temporary and will be replaced by next priority:  Perception that initiative is temporary and will eventually be replaced by other priorities | 5b. Organisation change readiness theory (14), social influence (17) | 5b. Gaming the measurement process:  Action taken to improve measurement process (e.g. self-selecting notes for audit) but no change to clinical care | *No impact*  Why was that the case?  (We’ve heard there’s lots of changes that are quite temporary…) |

COM-B = Capability, Opportunity, Motivation-Behaviour; TDF = Theoretical domains Framework); Green = positive outcome and Red = negative outcome

**References**

1. Michie S, van Stralen MM, West R. The behaviour change wheel: A new method for characterising and designing behaviour change interventions. Implementation Science. 2011;6(1):42.

2. Michie S, Johnston M, Abraham C, Lawton R, Parker D, Walker A. Making psychological theory useful for implementing evidence based practice: a consensus approach. Quality and Safety in Health Care. 2005;14(1):26-33.

3. Cane J, O’Connor D, Michie S. Validation of the theoretical domains framework for use in behaviour change and implementation research. Implementation Science. 2012;7(1):37.

4. Kluger A, DeNisi A. The Effects of Feedback Interventions on Performance: A Historical Review, a Meta-Analysis, and a Preliminary Feedback Intervention Theory. Psychological Bulletin. 1996;119:254-84.

5. Chaiken S, Ledgerwood A. A theory of heuristic and systematic information processing. Handbook of theories of social psychology, Vol 1. Thousand Oaks, CA: Sage Publications Ltd; 2012. p. 246-66.

6. Aronson E. The theory of cognitive dissonance: A current perspective. Advances in experimental social psychology. 4: Elsevier; 1969. p. 1-34.

7. Aronson J, Cohen G, Nail PR. Self-affirmation theory: An update and appraisal. 1999.

8. Kislov R, Wilson P, Cummings G, Ehrenberg A, Gifford W, Kelly J, et al. From Research Evidence to “Evidence by Proxy”? Organizational Enactment of Evidence-Based Health Care in Four High-Income Countries. Public Administration Review. 2019;79(5):684-98.

9. Stafford JE, Cocanougher BA. Reference group theory. Selected aspects of consumer behavior. 1977:361-80.

10. Lee TW, Locke EA, Latham GP. Goal setting theory and job performance. 1989.

11. Suls J, Wheeler L. Social comparison theory. Handbook of theories of social psychology. 2012;1:460-82.

12. Eagly AH, Chaiken S. Cognitive theories of persuasion. Advances in experimental social psychology. 17: Elsevier; 1984. p. 267-359.

13. Berkowitz AD. Applications of social norms theory to other health and social justice issues. The social norms approach to preventing school and college age substance abuse: A handbook for educators, counselors, and clinicians. 2003;1.

14. Weiner BJ. A theory of organizational readiness for change. Implementation science. 2009;4(1):1-9.

15. Hogg MA. Social identity theory: Springer; 2016.

16. Wood D. Foucault and panopticism revisited. Surveillance & Society. 2003;1(3):234-9.

17. Friedkin NE. A structural theory of social influence: Cambridge University Press; 1998.
